# Matcha as a Source of Bioactive Compounds: A Review of Health-Promoting Properties and Potential Applications

**DOI:** 10.3390/nu18101613

**Published:** 2026-05-19

**Authors:** Paulina Sławińska, Ewa Raczkowska

**Affiliations:** Department of Human Nutrition, Faculty of Biotechnology and Food Science, Wrocław University of Environmental and Life Sciences, 37 Chełmońskiego Street, 51-630 Wrocław, Poland; pslawinska.dietetyk@gmail.com

**Keywords:** matcha, green tea, EGCG, L-theanine, catechins, health-promoting properties

## Abstract

Matcha, a finely milled powdered green tea originating from Japan, is characterized by a unique cultivation method in which tea plants are shaded prior to harvest. This practice enhances the accumulation of chlorophyll, caffeine, L-theanine, and other bioactive compounds. In addition, specialized post-harvest processing, including careful hand-picking, gentle steaming, drying, and traditional stone grinding, helps preserve the nutritional and biochemical integrity of the tea leaves. This review examines the relationship between cultivation and processing techniques and the resulting bioactive composition of matcha. It also summarizes current scientific evidence regarding the potential health-promoting properties of matcha and its major constituents. The analysis is based on available scientific literature, including both in vitro and in vivo studies investigating the biological activity of matcha and green tea catechins. Particular attention is given to studies evaluating their effects on metabolic parameters such as glucose levels, lipid profile, body weight regulation, and gut microbiota composition. In addition, the potential influence of matcha-derived compounds on neurological function, systemic physiological processes and anticancer potential is discussed. Furthermore, matcha is increasingly recognized as a functional food ingredient and has been incorporated into a variety of products, including bakery goods, dairy products, functional beverages, and nutraceutical formulations. The collected findings suggest that matcha may exert a broad spectrum of beneficial biological effects due to its high concentration of polyphenols, amino acids, and antioxidants. Nevertheless, despite promising experimental and preclinical data, further well-designed clinical studies are needed to better understand the mechanisms of action, bioavailability, and long-term health effects associated with regular matcha consumption.

## 1. Introduction

Tea is one of the most popular beverages in the world. Green tea was the first type of tea ever discovered [1]. It is obtained from the leaves of the *Camellia sinensis* plant. Green tea is available on the market in three main forms: loose leaves, tea bags, and powdered leaves (matcha) [2]. Matcha is powdered green tea made from the Tencha variety [3]. It was once most popular in Japan; however, in recent years, its use in the food industry has increased significantly [3]. Recently, there has been a significant rise in the global popularity of matcha tea, mainly due to its distinctive taste and vibrant color [4,5]. Numerous recipes featuring matcha can be found online, and many cafés now offer various flavored versions of this beverage. The difference between matcha and sencha, aside from cultivation methods, is in the way it is consumed—matcha involves drinking the entire finely ground leaf suspended in water, whereas traditional green tea (sencha) is prepared as an infusion. As a consequence, matcha consumption results in the ingestion of the entire leaf matrix [5]. Because matcha is produced from shade-grown leaves and consumed in powdered form, it may provide higher amounts of amino acids and bioactive compounds responsible for its distinctive flavor and characteristic green color [6]. The unique compositional profile of matcha may translate into distinct biological effects, including enhanced antioxidant capacity, anti-inflammatory activity, and potential benefits in lipid metabolism [7,8]. Therefore, understanding differences in the concentration of individual constituents is essential when interpreting experimental and clinical findings. The differences in the content of individual components are presented in Table 1. It should be noted that the values presented are illustrative examples derived from selected studies and do not represent fixed concentrations for all matcha or sencha products. Both matcha and sencha are not uniform products, and their composition may vary depending on quality grade, cultivation conditions, and processing methods. Higher-grade matcha is typically characterized by a greater concentration of bioactive compounds, including caffeine and amino acids, particularly L-theanine, whereas lower-grade matcha may contain higher catechin concentrations which may influence its biological effects [9].

To prepare matcha in the traditional way, several tools are required: chashaku—a bamboo scoop used to measure the correct amount of matcha, chawan—a bowl in which the tea is prepared, and chasen—a bamboo whisk used to properly mix and froth the matcha. For the preparation of a serving of matcha, approximately 2 g of matcha powder is typically used with about 90–100 mL of water [6,10]. It should be prepared with hot (70 °C) but not boiling water to preserve its properties [11]. Different quality categories of matcha can be distinguished. The highest quality is ceremonial-grade matcha, produced from buds and the first leaves harvested in spring, when they are more tender—this directly affects the flavor profile. The later the harvest, the older and larger the leaves, resulting in a less delicate taste typical of culinary-grade matcha. The next steps in production include steaming, drying, and cooling, followed by trimming stems, removing veins, petioles, and older leaves. Ceremonial matcha is ground using traditional stone mills, which is a slow process. Faster grinding methods are used for lower-quality matcha [9].

Understanding the impact of cultivation and processing methods on matcha quality is essential for interpreting its biological activity and potential health benefits. In light of the expanding body of scientific literature, a comprehensive synthesis of current findings is warranted. Therefore, the aim of this review is to present and critically evaluate available evidence on the composition of matcha and its documented physiological effects, highlighting both its therapeutic potential and existing research gaps. In addition, this review addresses the growing interest in the application of matcha as a functional food ingredient, emphasizing its use in food systems and nutraceutical formulations.

While several review articles have addressed the health effects of green tea or selected aspects of matcha, a comprehensive synthesis integrating cultivation conditions, biochemical composition, and mechanistic links to biological activity remains limited [12,13].

Therefore, this review aims not only to summarize current evidence on the health-promoting properties of matcha but also to: highlight the impact of cultivation and processing methods on its bioactive profile, link specific compounds with their biological effects and discuss its emerging role as a functional food ingredient. In addition, this review incorporates recent findings from both human and experimental studies published between 2016 and 2025, providing an updated and integrative perspective.

**Table 1:** Content of individual components in matcha tea and sencha.

	Matcha (Portion ~2 g)	Sencha (Portion ~235 mL)
Total catechins	170 mg [14]	50.99–160.15 mg [15]
Epigallocatechin gallate (EGCG)	109.8 mg [14]	10–80 mg [16]
Epicatechin gallate (ECG)	20.3 mg [14]	42.063 mg [17]
Epigallocatechin(EGC)	33.1 mg [14]	15.69–55.60 mg [15]
Epicatechin (EC)	8.3 mg [14]	19.505 mg [17]
Gallocatechin gallate (GCG)	1.0 mg [14]	-
Catechin gallate (CG)	0.1 mg [14]	[17]
Gallocatechin (GC)	1.4 mg [14]	3.07–8.58 mg [15]
Catechin (C)	0.8 mg [14]	0–1.99 mg [15]
L-theanine	50.3 mg [14]	-
Caffeine	72.5 mg [14]	28.2–37.6 mg [18]

## 2. Article Search Methodology

This review was based on scientific literature published in the English language. The following electronic databases were used to identify relevant studies: PubMed, Web of Science, Scopus and Google Scholar. The search strategy included combinations of the following keywords: matcha OR matcha green tea AND cholesterol OR lipid profile OR triglycerides OR glucose level OR insulin level OR liver OR microbiota OR body weight OR inflammation OR cancer OR health OR cognitive function. The manual selection of literature entries was sufficient to obtain an adequate amount of data.

Articles used in this review were published between 2016 and 2025. To prepare tables presenting the biological effects of matcha, 19 articles were used; human intervention studies—9; in vivo studies—5; and in vitro studies—5.

## 3. Macronutrient Composition of Matcha Powder

Chemical analyses examining the composition of matcha powder have demonstrated that 100 g of the product contains approximately 17.3 g of plant-derived protein. The fat content is about 7.285 g/100 g, of which more than 83% consists of unsaturated fatty acids. Among these, omega-3 fatty acids, particularly α-linolenic acid, constitute the predominant fraction. The total dietary fibre content is 56 g/100 g, with 94.1% represented by insoluble fibre fractions [19]. This relatively high fibre content is directly related to the fact that matcha is produced from whole powdered tea leaves, which are consumed in their entirety. In contrast to sencha—where the leaves are steeped and subsequently discarded—matcha provides the full leaf matrix, resulting in a substantially higher intake of fibre and other insoluble constituents [5].

### The Effect of Cultivation Method on Biochemical Composition

Tea leaves intended for matcha production are grown in shaded areas [2]. Shaded cultivation increases the levels of chlorophyll, caffeine, and L-theanine in the leaves, while decreasing the content of epicatechin, epicatechin gallate, epigallocatechin, and epigallocatechin gallate [5,20]. Moderate shading may also lead to an increase in the amino acid content, which correlates positively with the market value of matcha. However, excessive shading (95%) may have the opposite effect [5]. The biochemical changes observed in shade-grown tea leaves can be explained by alterations in plant metabolic pathways. Reduced light exposure limits photosynthetic activity, which decreases the conversion of amino acids into polyphenolic compounds such as catechins. As a result, nitrogen-containing compounds, particularly amino acids such as L-theanine, accumulate in higher concentrations. At the same time, shading promotes chlorophyll synthesis as an adaptive response to low light conditions, enhancing light-harvesting efficiency and contributing to the characteristic intense green color of matcha [21].

In addition, shading affects the balance between primary and secondary metabolism. Under reduced light conditions, the plant shifts toward nitrogen metabolism and amino acid retention, while the biosynthesis of certain flavonoids and catechins may be relatively suppressed. This metabolic shift contributes to the milder taste and umami profile of matcha compared to non-shaded green tea [22,23]. Toniolo et al. compare metabolite profiles among different types of matcha, ranging from high-quality ceremonial to culinary-grade varieties. The amino acid concentration decreased with lower tea quality; a phenomenon related to leaf aging [9]. Leaf age is another critical factor influencing the distribution of bioactive compounds. Younger leaves are metabolically more active and serve as primary sites of growth, which leads to preferential accumulation of amino acids such as L-theanine. These compounds are actively transported from older tissues to developing leaves, where they support growth and stress responses. In contrast, older leaves tend to accumulate higher levels of secondary metabolites, including catechins and flavonoids, which play a protective role against environmental stressors such as UV radiation and herbivory [24,25].

This differential distribution reflects a trade-off between growth-related metabolism in young tissues and defense-related metabolism in mature leaves, which explains the compositional differences observed between high-quality (young leaf) and lower-grade (older leaf) matcha. This was most evident for theanine—the most abundant amino acid—present in the highest amounts in young leaves. As leaves age, theanine is transported to younger parts of the plant. Similar observations were made for caffeine—young leaves contain higher levels compared to older ones, as caffeine serves a protective role against phytophagous organisms in young plant tissues. The content of organic acids, such as chlorogenic acid and shikimic acid, also decreased as the leaves matured. Conversely, the concentration of flavonoids—kaempferol and rutin—was lowest in young leaves and increased proportionally with leaf age. The catechin content was high overall, which is characteristic of green tea; however, samples of lower-quality matcha exhibited the highest catechin levels, reflecting the accumulation of these metabolites in older leaves [9].

The harvest season of tea leaves also affects the fluoride content of matcha. One litre of matcha tea may provide up to 4 mg of fluoride. This estimate is based on a typical preparation ratio of 1.75 g of matcha powder per 100 mL of water (i.e., 17.5 g per litre). According to the European Food Safety Authority (EFSA), the adequate intake (AI) for fluoride is defined as 0.05 mg per kilogram of body weight per day, corresponding to 3.5 mg/day for a 70 kg adult [26]. In a study by Jakubczyk et al., matcha produced from leaves collected during the second and third harvests (daily) exhibited a higher fluoride concentration than matcha derived from the first and second harvests (traditional). Additionally, fluoride content increases with the temperature of the water used for preparation [26]. This is attributable to increased fluoride extraction with rising temperature [27]. Importantly, matcha is conventionally prepared using water at 70 °C instead of boiling water, which could moderate the degree of fluoride extraction under typical consumption conditions [11]. This factor should be considered to avoid excessive accumulation of fluoride in the body and potential adverse effects. However, traditional consumption typically involves one to two servings per day (approximately 1.75–2 g of matcha per serving, prepared with 70 °C water), which would provide substantially less fluoride than one litre consumed at once. Under such customary intake patterns, fluoride exposure from matcha alone would generally remain within the adequate intake range for adults, although total dietary fluoride intake from other sources should also be taken into account.

## 4. Health-Promoting Properties of Matcha

The biological effects of matcha are primarily attributed to its major bioactive constituents, including catechins (especially epigallocatechin gallate, EGCG), L-theanine, caffeine, and other polyphenols. These compounds exert diverse biological activities through multiple molecular mechanisms.

Catechins, particularly EGCG, are responsible for strong antioxidant and anti-inflammatory effects and may modulate key signaling pathways such as AMPK (AMP-activated protein kinase), PI3K/Akt, and mTOR [18]. L-theanine contributes mainly to neuroprotective and cognitive effects through modulation of neurotransmitters, including GABA, dopamine, and serotonin. Caffeine, in turn, influences central nervous system activity by antagonizing adenosine receptors, leading to increased alertness and enhanced energy metabolism [28].

Importantly, these compounds may act synergistically, and their combined effects are likely responsible for the broad spectrum of physiological outcomes associated with matcha consumption.

The health-promoting properties of matcha have attracted increasing scientific interest due to its high content of biologically active compounds such as catechins, L-theanine, and other polyphenols. These constituents have been associated with a range of physiological effects, including effects on carbohydrate metabolism and lipid profiles, as well as potential anticancer properties. In this review, sections are organized according to the specific biological effects attributed to matcha consumption, allowing for a detailed discussion of its potential mechanisms of action and physiological outcomes. However, because the number of studies directly examining certain health effects of matcha-such as its influence on glucose metabolism, lipid profile, or body weight regulation-remains relatively limited, the available evidence is heterogeneous and derived from different experimental models. Therefore, the studies summarized in the tables are classified according to the type of research design. Human intervention studies evaluating the biological effects of matcha or matcha-derived bioactive compounds are summarized in Table 2, while evidence from in vivo studies is presented in Table 3. This classification provides a clearer overview of the current state of research and facilitates comparison between different experimental approaches, while the following subsections discuss the reported health effects in greater detail.

### 4.1. Cognitive Function and Sleep

The effects of matcha on cognitive function are primarily attributed to the synergistic interaction between L-theanine and caffeine. L-theanine has been shown to promote relaxation without sedation by modulating neurotransmitters such as GABA (gamma-aminobutyric acid), dopamine, and serotonin, while caffeine enhances alertness through antagonism of adenosine receptors. Additionally, catechins, particularly EGCG, may exert neuroprotective effects through antioxidant activity and reduction in neuroinflammation [28]. Matcha tea has been widely investigated for its effects on cognitive function, brain activity, and neuroprotection. In a 12-month study by Uchida et al. (2024) [10], the authors assessed the effects of matcha on cognitive performance and sleep quality in older adults with subjective or mild cognitive impairment—conditions often associated with impaired emotion perception and facial expression recognition. The study demonstrated an improvement in social cognition following matcha consumption. Participants in the intervention group also showed improved scores on the Pittsburgh Sleep Quality Index (PSQI), suggesting a potentially beneficial effect of matcha on sleep quality. The daily portion of matcha used in the study contained 48.1 mg of theanine, 170.8 mg of catechins, including 105.3 mg of EGCG, and 66.2 mg of caffeine [10]. In contrast, a 4-week intervention study found no significant changes in sleep duration, sleep latency, wake after sleep onset, or sleep efficiency; however, participants consuming matcha reported a subjective improvement in sleep quality. The daily portion of matcha used in the study contained 50.3 mg of theanine, 301.5 mg of catechins, including 143 mg of EGCG, and 71.5 mg of caffeine [28]. The authors suggested that these effects may be attributable to L-theanine contained in matcha [10,28].

Nevertheless, further research focusing specifically on REM and NREM sleep architecture is lacking. Additionally, factors such as genetically determined caffeine sensitivity warrant investigation.

### 4.2. Human Microbiota

The modulatory effects of matcha on gut microbiota are largely associated with its high content of polyphenols, especially catechins such as EGCG. These compounds are poorly absorbed in the small intestine and therefore reach the colon, where they interact with gut microorganisms. Catechins may promote the growth of beneficial bacteria while inhibiting pathogenic species, and their microbial metabolites can further influence host metabolism and inflammation [30].

In a study by Morishima et al. (2023) [30], the effects of matcha consumption on the human fecal microbiota were evaluated. Fecal samples were collected before and after the intervention from participants consuming matcha and from a placebo group. Twice-daily matcha intake over two weeks resulted in a beneficial shift in microbial composition, including an increase in *Coprococcus* and a decrease in potentially pathogenic *Fusobacterium*. Moreover, a significant improvement in microbial diversity was observed in the matcha group [30]. Another study examining matcha intake in the context of physical exercise reported that the abundance of several gut bacterial genera increased after four weeks of consumption; however, these differences were no longer evident after eight weeks. The observed changes involved *Butyricimonas*, *Ruminococcus*, and *Oscillospira*. The daily portion of matcha used in the study contained 54 mg of theanine, 189 mg of EGCG, and 90 mg of caffeine [31]. In a study by Luo et al. (2024), conducted on mice, matcha was shown to potentially counteract obesity by modulating the gut microbiota, specifically by reducing bacteria associated with obesity, including *Alloprevotella*, *Ileibacterium*, *Rikenella*, and *Romboutsia* [36]. Chong et al. (2025) [37] similarly observed that dietary matcha supplementation increased the abundance of *Parabacteroides* and *Akkermansia*—microorganisms linked to the attenuation of obesity, metabolic syndrome, and diabetes—as well as *Faecalibacterium*, which may mitigate non-alcoholic fatty liver disease (NAFLD). The study was conducted in an animal model [37]. Wang et al. likewise reported an increase in *Akkermansia muciniphila*, alongside changes in microorganisms relevant to NAFLD, such as an increase in *Alloprevotella* (a short-chain fatty acid producer whose depletion is associated with NAFLD) and suppression of *Mucispirillum schaedleri*, considered NAFLD-associated, in a mouse model [38]. It should be noted that the studies by Luo et al. (2024), Chong et al. (2025), and Wang et al. (2022) were conducted in animal models [36,37,38]. Therefore, further research is required to determine the extent to which matcha may modulate human gut microbiota and, through this mechanism, influence conditions such as obesity and NAFLD.

### 4.3. Body Weight

The potential effects of matcha on body weight regulation are mainly linked to catechins (particularly EGCG) and caffeine. EGCG has been shown to enhance fat oxidation and energy expenditure, partly through activation of AMP-activated protein kinase (AMPK), while caffeine may further stimulate thermogenesis and lipolysis. These combined effects may contribute to increased fat utilization, particularly during physical activity [18,36].

Clinical studies have shown that matcha consumption may increase fat oxidation rates during exercise (*p* < 0.01), suggesting a potential role as an adjunct for body weight management. In this study premium-grade matcha was used [29]. However, interventional findings indicate that these effects are moderate and influenced by coexisting factors such as dietary patterns, habitual physical activity, and individual metabolic characteristics. Additional randomized controlled trials are needed to more clearly define matcha’s role in weight control [32]. Preclinical (animal) studies demonstrate that regular administration of matcha may attenuate body weight gain and adipose tissue accumulation in animals fed a high-fat diet. These effects have been attributed to increased energy expenditure, modulation of gut microbiota, improved lipid metabolism, and reduced inflammation. Although promising, these results require confirmation in rigorously designed human trials to establish matcha’s efficacy and mechanisms in weight management [36,38].

### 4.4. Glucose and Insulin Level

The influence of matcha on glucose metabolism is primarily attributed to catechins, especially EGCG, as well as chlorogenic acid. These compounds may improve insulin sensitivity, reduce intestinal glucose absorption, and modulate incretin hormones such as GLP-1. Additionally, antioxidant and anti-inflammatory effects may contribute to improved metabolic regulation [33].

Research on the effects of matcha and other forms of green tea on glucose metabolism and insulin regulation is expanding, although results remain mixed.

In a crossover study conducted in healthy men, a three-week supplementation with a mixture of green tea catechins and chlorogenic acids significantly improved postprandial glycemic response, increased GLP-1, and decreased GIP concentrations compared with placebo [33]. Although matcha was not the exclusive source of catechins, the findings highlight the potential metabolic relevance of catechin intake. A meta-analysis of 27 randomized trials (2194 participants) showed that green tea supplementation reduced fasting glucose by an average of −1.44 mg/dL (95% CI: −2.26, −0.62 mg/dL; *p* < 0.001) but had no effect on fasting insulin or HbA1c [40]. Another systematic review and meta-analysis of 14 studies found no significant effect of green tea consumption on fasting glucose, fasting insulin, HbA1c, or HOMA-IR in individuals with type 2 diabetes [41]. Although studies specifically on matcha are more limited, a pilot trial involving overweight/obese adults reported a trend toward reductions in glucose and insulin after a 12-week matcha intervention, though the differences between the intervention and control groups did not reach statistical significance [32].

In summary, current evidence suggests that matcha or sencha consumption may modestly reduce fasting glucose, improve postprandial glycemic response, and—in some cases-enhance insulin sensitivity. However, the magnitude of these effects is generally small, and no clear benefits have been established for individuals with type 2 diabetes. Interpretation is further limited by substantial heterogeneity across studies (including variations in dose, form, and duration) and by the scarcity of research focused specifically on matcha.

### 4.5. Lipid Profile and Liver

The beneficial effects of matcha on lipid metabolism and liver function are largely associated with catechins, particularly EGCG, which may regulate lipid metabolism through activation of AMPK and inhibition of lipogenesis. Catechins may also reduce oxidative stress and inflammation in hepatic tissue, thereby limiting lipid accumulation and improving liver enzyme profiles [8,18].

The following section summarizes evidence from animal models investigating the effects of matcha on lipid profile and liver parameters. In their study, Xu et al. investigated how matcha consumption affects the lipid profile and metabolic parameters in mice fed a high-fat diet. The tested interventions included matcha powder, a matcha aqueous extract, and the residues remaining after extraction. It was observed that in mice receiving a high-fat diet supplemented with medium and high doses of matcha, total cholesterol levels were lower than in mice fed a high-fat diet without supplementation. Matcha also reduced triglyceride levels, both in the blood and in the liver [39].

When individual cholesterol fractions were analyzed, the greatest increase in HDL cholesterol was observed in mice fed a high-fat diet supplemented with medium and high doses of matcha, as well as in those receiving the extraction residues. In the same groups, the lowest LDL cholesterol levels were also reported compared with mice fed a high-fat diet without supplementation [39]. Zhou et al. likewise reported reductions in TG and TC levels, as well as a decreased LDL/HDL ratio, in the matcha-supplemented group. Moreover, decreased ALT and AST activities were observed in the matcha group—enzymes commonly regarded as biochemical indicators of the extent of liver injury. In mice fed a high-fat diet, excessive lipid accumulation and inflammatory cell infiltration were detected, whereas in animals whose diet was enriched with matcha, only very mild steatosis and limited inflammatory foci were observed. The matcha group also exhibited reduced hepatic levels of the inflammatory biomarkers TNF-α, IL-6, and IL-1β, which were markedly elevated in the HFD (high-fat diet) group compared with the control group [8].

### 4.6. Oral Health

The effects of matcha on oral health are primarily related to its high content of catechins and other polyphenols, which exhibit antibacterial and antioxidant properties. EGCG, in particular, has been shown to inhibit the growth of oral pathogens such as *Porphyromonas gingivalis* and *Streptococcus mutans*, while also reducing oxidative stress in gingival tissues [34,35].

Further research is required to precisely evaluate the impact and clinical effectiveness of matcha in the context of oral health; however, several preliminary findings suggest potential beneficial effects. These effects are primarily attributed to matcha’s high antioxidant content.

In a study by Abood et al., the influence of matcha consumption on patients with localized gingivitis was assessed. Daily intake of matcha, consumed twice per day, improved gingival condition and reduced markers of oxidative stress [34]. Another study evaluated gingival and periodontal health following one month of mouth-rinsing with a matcha extract. A significant reduction in gingival and periodontal inflammation was observed, with effects comparable to those achieved with a 2% chlorhexidine gluconate rinse. Moreover, in vitro experiments demonstrated the efficacy of matcha extract in inhibiting *Porphyromonas gingivalis* and *Streptococcus mutans* [35]. Nevertheless, given the short duration of the intervention, further long-term studies are warranted. Moreover, chlorhexidine use is known to be associated with several side effects, such as extrinsic tooth staining, taste alteration and xerostomia, which may limit its prolonged application [42].

### 4.7. Anticancer Potential

The anticancer potential of matcha is mainly attributed to catechins, especially EGCG, which has been extensively studied for its ability to modulate key cellular pathways. EGCG may induce apoptosis, inhibit cell proliferation, and interfere with signaling pathways such as PI3K/Akt, mTOR, and MAPK (mitogen-activated protein kinase). Additionally, its antioxidant and epigenetic effects may contribute to the regulation of gene expression in cancer cells [18]. Matcha exhibits notable anticancer potential, modulating key processes involved in tumor development and progression. Findings from recent investigations—including both cellular and animal models—highlight the multifaceted effects of matcha extracts on cancer cell proliferation, metabolism, and survival.

One of the most promising areas concerns matcha’s influence on cell-cycle regulation and the induction of apoptosis. In a study using the human oral squamous cell carcinoma line SCC-15, matcha extract significantly reduced cancer cell viability and increased the proportion of cells in late apoptosis. These effects were accompanied by upregulation of the pro-apoptotic protein p53 and downregulation of the anti-apoptotic protein Bcl-2, indicating activation of intrinsic apoptotic pathways [43].

Research on breast cancers—including the particularly aggressive triple-negative subtype (TNBC)—further supports the ability of matcha to inhibit tumor growth and limit metastasis. In a zebrafish model, exposure to a properly prepared matcha extract reduced tumor size and decreased the number of cancer cells derived from human TNBC lines in a dose-dependent manner. In the study, organic ceremonial-grade matcha was used [44].

Another important aspect of matcha’s anticancer activity is its effect on highly proliferative cell populations. Studies in the MCF7 breast cancer line demonstrate that matcha markedly suppresses the formation of cellular spheroids, a recognized indicator of cancer stem-cell activity. Proteomic analyses simultaneously revealed broad disruptions in mitochondrial metabolism (OXPHOS), glycolysis, and mTOR signaling, suggesting metabolic reprogramming of cancer cells that limits their growth and adaptive capacity [45]. In another study focusing on breast cancer, a beneficial effect of matcha was also demonstrated. Following stimulation of MCF7 cells with matcha tea extract, a reduction in cell viability and a decrease in Erβ protein levels were observed. However, further studies are required to precisely determine the mechanisms underlying the reported effects of matcha in the context of cancer cells and estrogen receptor expression [46]. The study by Keckstein et al. on T47D cells also addressed the potential of matcha in the context of breast cancer. Similarly, this study reported reduced cell viability and increased PPARγ expression at both the protein and mRNA levels [47].

Collectively, the available experimental data strongly indicate that matcha possesses anticancer potential encompassing inhibition of cancer cell proliferation, induction of apoptosis, suppression of cancer stem-cell–like properties, and disruption of metabolic and signaling pathways. However, due to the absence of clinical studies and substantial variability among matcha preparations, matcha cannot currently be recommended as an effective anticancer intervention in humans. These findings should be viewed as promising preliminary evidence that warrants rigorous confirmation—particularly in well-designed translational and clinical research.

In vitro studies describing the molecular mechanisms of matcha and sencha bioactive compounds are summarized in Table 4.

## 5. Potential Applications of Matcha as a Functional Food Ingredient

Matcha is increasingly recognized not only as a traditional beverage but also as a versatile ingredient in the development of functional foods. Its high content of bioactive compounds, including amino acids, polyphenols, chlorophyll, L-theanine, and caffeine, contributes to its growing application across diverse food matrices [7,12].

In bakery products, matcha has been extensively investigated as a functional additive in bread, cakes, cookies, and gluten-free formulations. Its incorporation has been shown to enhance antioxidant capacity, providing protection against oxidation, as well as improving the overall nutritional profile of final products [48]. However, technological parameters such as texture, porosity, volume, and sensory acceptability may be affected depending on the level of addition. Higher concentrations of matcha may also disrupt the gluten protein network structure, leading to alterations in product quality [49,50].

In dairy products and desserts, matcha is used as a natural source of bioactive compounds and pigments. It has been incorporated, for example, into ice cream, where it influenced the profile of organic acids, mineral composition, and sensory attributes. Moreover, its characteristic flavor and color may enhance product appeal, although formulation optimization is required to achieve a balanced sensory profile [51].

Matcha is also increasingly utilized in functional beverage formulations, including ready-to-drink teas, smoothies, and fortified beverages (e.g., protein-enriched products). In these applications, it serves as a natural antioxidant and a source of bioactive compounds [6].

Furthermore, matcha and its isolated bioactive compounds are gaining attention in the development of nutraceuticals and dietary supplements. Due to the limited stability and low bioavailability of catechins, such as epigallocatechin gallate (EGCG), various encapsulation and nanoencapsulation techniques, as well as advanced delivery systems-including protein-, lipid-, and carbohydrate-based carriers-are being explored to enhance their stability, enable controlled release, and improve intestinal absorption [52,53].

Despite these promising applications, further research is required to evaluate the stability of matcha-derived compounds during processing and storage, as well as their bioavailability and efficacy within complex food matrices.

## 6. Safety of Use

Available evidence indicates that, under moderate and typical dietary consumption of green tea infusions and extracts, the risk of adverse effects in healthy adults is low. However, certain safety concerns arise with the use of highly concentrated extracts or very high doses of catechins. The principal areas of risk include hepatotoxicity associated with high-dose extracts, drug–nutrient interactions, as well as potential exposure to heavy metals [17].

Systematic reviews and safety reports have documented cases of acute liver injury associated with the consumption of concentrated green tea extracts containing high levels of epigallocatechin gallate (EGCG). In its 2018 scientific opinion, the European Food Safety Authority (EFSA) emphasized evidence indicating a risk of hepatotoxicity from supplements delivering large doses of catechins and proposed a precautionary threshold for such products. It has been established that it is not possible to define a dose of EGCG from green tea extracts that can be considered universally safe. However, available evidence indicates that no adverse effects on liver function were observed at daily doses below 800 mg EGCG. Importantly, clinical case reports of liver injury linked to green tea intake have most frequently involved dietary supplements, rather than the traditional consumption of brewed tea or matcha powder in amounts typical of the diet [17,54].

It is hypothesized that high hepatic concentrations of EGCG (and/or its metabolites) may induce oxidative stress and hepatocellular damage in susceptible individuals. At high concentrations, EGCG may undergo autoxidation, forming reactive o-quinone derivatives, which can react with glutathione and subsequently form EGCG thiol conjugates [54,55]. In some cases, concomitant medication use or underlying metabolic predispositions may further increase this risk. Green tea should not be co-administered with drugs metabolized by CYP1A2, CYP2C9, and CYP3A4, as its catechins may inhibit the activity of these enzymes [56]. EGCG may also affect the pharmacokinetics of anticancer drugs. In a study investigating irinotecan, EGCG was shown to modulate the activity of the P-glycoprotein transporter, leading to alterations in the plasma concentrations of the drug and its active metabolite SN-38. Such interactions may potentially increase the risk of adverse effects or toxicity [57]. In summary, these mechanisms may lead to changes in the pharmacokinetics of both co-administered drugs and EGCG itself, which in some cases may increase the risk of adverse effects, including potential hepatotoxicity. Consequently, safety assessments should clearly distinguish between risks associated with standardized, concentrated supplements and those related to habitual consumption of beverages prepared from whole tea leaves [54].

Studies also suggest that compounds present in green tea, including catechins and other polyphenols, may alter the bioavailability or metabolism of certain medications. Reported interactions include reduced absorption of some statins, anticoagulants, and other drugs transported by organic anion-transporting polypeptides (OATPs) or related intestinal transporters. Clinically, this may result in reduced therapeutic efficacy (e.g., lower systemic drug concentrations) or, less frequently, increased toxicity in the context of specific interactions. Therefore, individuals taking medications with documented interactions should consult a healthcare professional regarding regular or high intake of green tea or matcha [58].

Because matcha is consumed as the entire powdered leaf rather than as an infusion, the concentration of potential contaminants—such as lead or cadmium—may be higher than in conventional brewed tea. Monitoring studies assessing metal levels in tea leaves and infusions have demonstrated variability depending on geographical origin and raw material quality. Regular consumption of low-quality products sourced from areas with contaminated soil may increase exposure. Accordingly, the selection of certified, high-quality products is recommended, and consumption of matcha from unknown or unverified sources should be limited [59].

## 7. Conclusions

Matcha has gained considerable scientific interest due to its rich composition of bioactive compounds, including catechins, caffeine, L-theanine, and other polyphenols, which may contribute to a range of health-promoting effects. Evidence from human intervention studies suggests that matcha and its bioactive components may exert beneficial effects particularly in the context of metabolic health, including improvements in glucose metabolism, lipid profile, and markers of inflammation. These findings are further supported by numerous in vivo and in vitro studies demonstrating potential mechanisms related to antioxidant activity, modulation of inflammatory pathways, and regulation of metabolic processes.

In clinical studies, the doses of matcha or matcha-derived compounds vary considerably, although most interventions typically involve daily intake equivalent to approximately 1.5–3 g of matcha powder or comparable amounts of bioactive constituents such as catechins. While several studies report beneficial effects, the overall evidence remains somewhat heterogeneous, partly due to differences in study design, intervention duration, and the form of the investigated product (matcha powder, green tea extracts, or isolated compounds).

The quality of available studies also varies. Some human trials are randomized and controlled, which strengthens the evidence base; however, many studies involve relatively small sample sizes and short intervention periods. Additionally, the standardization of matcha preparations is often limited, making comparisons between studies more difficult. Variability in study populations and experimental models further contributes to inconsistencies in the reported outcomes.

A limitation in interpreting the available evidence is also the lack of consistent reporting on the quality or grade of matcha used in the analyzed studies. In many cases, studies do not specify whether ceremonial or culinary-grade matcha was used, which may affect the concentration of key bioactive compounds such as catechins and caffeine. Therefore, differences in matcha quality may contribute to variability in the reported results and should be taken into account when comparing findings across studies.

Despite these limitations, the current body of evidence suggests that matcha may have potential as a functional food with possible benefits for metabolic health and overall well-being. Regular consumption of matcha could therefore be considered as a supportive dietary component within strategies aimed at the prevention of metabolic disorders. Nevertheless, further well-designed, long-term randomized controlled trials with standardized matcha preparations and larger study populations are needed to confirm these findings and to establish optimal dosing strategies.

This review provides an integrative perspective linking cultivation practices, chemical composition, and biological activity of matcha, while also highlighting its potential applications in functional foods and identifying key gaps in current research.

## Figures and Tables

**Table 2:** Human intervention studies on the biological effects of matcha or matcha-derived bioactive compounds.

Study Population	Intervention (Form/Dose)	Exposure Time	Main Observed Effects	Source
13 females	3 × 1 g of matcha mixed with water on the day before testing, matcha tea prior to exercise (1 g of matcha per drink)	Before exercise	↑ fat oxidation during exercise; no effect on maximal performance	[29]
99 older adults with cognitive impairment	Matcha tea (2 g of matcha) per day	12 months	↑ social cognition, ↑ PSQI	[10]
Healthy Japanese men and women aged 27–64 years	2.7 g matcha daily (15 capsules per day)	4 weeks	↑ subjective sleep quality, ↑ emotional stability	[28]
33 participants	Matcha tea (1.5 g of matcha) twice a day	2 weeks	↑ *Coprococcus*, ↓ *Fusobacterium*, ↑ the beta-diversity of microbial composition	[30]
36 healthy, untrained men	Matcha tea (1.5 g of matcha) twice a day	8 or 12 weeks	accelerating muscle adaptation to resistance training; positive correlations between changes in muscle adaptation and microbiota	[31]
40 participants	Low-calorie diet + matcha tea per day (2 g of matcha)	12 weeks	↑ HDL-C, ↓ blood glucose, ↑ HbA1c, ↓ insulin and leptin levels, ↑ the activity of superoxide dismutase, ↓ activity of glutathione peroxidase, ↑ IL-10	[32]
11 healthy men	GTC + CCA-enriched beverage (620 mg GTC, 373 mg CCA and 119 mg caffeine per day)	3 weeks	Glucose changes after the ingestion of high-fat and high-carbohydrate meals but it did not affect fasting glucose levels, ↑ postprandial insulin sensitivity ↑ postprandial GLP-1, ↓ GIP	[33]
27 participants with localised gingivitis	Matcha tea twice a day	30 days	↓ inflammation of the gingival tissue, ↓ PI, BOP, 8-OHdG, ↓ bleeding	[34]
32 adult patients having mild-to-moderate gingivitis and periodontitis	Matcha mouthrinse; rinsing twice a day for 1 min	28 days	The matcha mouthrinse exhibits an effect similar to that of 2% CHX gluconate; ↓ the inflammatory condition of gingivitis and periodontitis, ↓ *P. gingivalis* and *S. mutans*	[35]

Abbreviations: PSQI, Pittsburgh Sleep Quality Index; HDL-C, high-density lipoprotein cholesterol; HbA1c, hemoglobin A1c; IL-10, interleukin 10; GTC, green tea catechins; CCA, coffee chlorogenic acids; GLP-1, glucagon-like peptide-1; GIP, glucose-dependent insulinotropic polypeptide; PI, plaque index; BOP, bleeding on probing; 8-OHdG, 8-hydroxy-deoxyguanosine; CHX, chlorhexidine; ↓, decrease; ↑, increase.

**Table 3:** In vivo studies on the biological effects of matcha and sencha.

Study Population	Intervention (Form/Dose)	Exposure Time	Main Observed Effects	Source
30 C57BL/6J male mice (6 weeks old)	Matcha physiological saline solution (1 g/kg body weight)	17 weeks	↓ deleterious effects of a high-fat diet on body weight, adipose tissue weight, and lipid levels, ↑ glucose tolerance in obese mice, ↑ *Alloprevotella*, *Ileibacterium*, *Rikenella*, ↓ *Romboutsia*, ↑ the caffeine metabolism and HIF-1 signaling pathways in KEGG metabolic pathways	[36]
48 male Wistar rats (7-week-old)	0.2% (g/g) matcha powder ~1.2 g matcha powder for humans (half a cup of matcha tea), 1% (g/g) matcha powder ~6 g matcha powder for humans (3 cups of matcha tea)	12 weeks	↓ triglycerides, blood sugar, serum insulin, HOMA-IR, plasma leptin, hepatic cytokines, ↑ *Akkermansia*, *Faecalibacterium*, *Parabacteroides*	[37]
20 male C57BL/6J mice (7–8 weeks old)	1.0% matcha in diet	8 weeks	↓ the development of obesity, lipid accumulation, and hepatic steatosis induced by high fat diet, ↑ *Faecalibaculum*, *Alloprevotella*, *Akkermansia muciniphila*	[38]
70 male ICR mice	High fat diet with: - 0.025% matcha, - 0.05% matcha, - 0.075% matcha, - 0.05% matcha extract, - 0.05% matcha residues	4 weeks	↓ TC, TG, LDL-C, blood glucose level, MDA, ↑ HDL-C, SOD, GSH-Px	[39]
25 male C57BL/6 mice (4-week-old)	High fat diet blending with: - 0.1% matcha, - 0.5% matcha, - 1.0% matcha	6 weeks	↓ TG, TC, LDL/HDL, blood glucose, ALT, AST, TNF-α, IL-6, IL-1β	[8]

Abbreviations: HIF-1, hypoxia-inducible factor 1; KEGG, Kyoto encyclopedia of genes and genomes; HOMA-IR, homeostatic model assessment of insulin resistance; TC, total cholesterol; TG, triglyceride; LDL-C, low-density lipoprotein cholesterol; MDA, malondialdehyde; HDL-C, high-density lipoprotein cholesterol; SOD, superoxide dismutase; GSH-Px, glutathione peroxidase; ALT, alanine aminotransferase; AST, aspartate aminotransferase; TNF-α, tumor necrosis factor alpha; IL-6, interleukin-6; IL-1β, interleukin-1 beta; ↓, decrease; ↑, increase.

**Table 4:** In vitro studies on molecular mechanisms of matcha and sencha bioactive compounds.

Cell Culture	Intervention (Form/Dose)	Exposure Time	Main Observed Effects	Source
TSSC cell line (SCC-15)	Ethanolic MGT extract	IC50 values of MGT extract for different time intervals (24, and 48 h)	MGT extract is toxic to SCC-15. ↓ cell viability, ↑ apoptosis, ↑ p53 protein expression, ↓ Bcl-2 apoptotic genes expression	[43]
Triple-negative breast cancer cells (MDA-MB-468 and MDA-MB-231)	Two matcha extracts—50 µg/mL and 100 µg/mL; prepared in Embryo medium	2 days	↓ the tumor mass size of MDA-MB-231 cancer cells, ↓ the metastasis of the tumor cells	[44]
MCF7 cells (an ER(+) human breast cancer cell line)	MGT 0.2 mg/mL (dissolve in water)	72 h	↓ the propagation of breast cancer stem cells, ↓ glycolytic rate, ↓ OCR and ECAR, ↓ expression of specific mitochondrial proteins and glycolytic enzymes, ↑ p53 tumour suppressor protein and RB1	[45]
MCF7 cells	Ethanolic MTE 5 µg/mL and 10 µg/mL	72 h	↓ cell viability of MCF7 cells, ↓ Erβ at protein level	[46]
T47D cells	Ethanolic MTE 5, 10 and 50 µg/mL	72 h	↓ cell viability of T47D cells, ↑ expression of PPARγ in T47D cells	[47]

Abbreviations: TSSC, tongue squamous cell carcinoma; SCC-15, TSSC cell line; MGT, matcha green tea; MTE, matcha green tea extract; IC50, half maximal inhibitory concentration; MCF7, breast cancer cell line; ER, estrogen receptor; OCR, oxygen consumption rate; ECAR, extracellular acidification rate; RB1, tumor suppressor retinoblastoma protein; Erβ, estrogen receptor-beta; T47D, breast cancer cell line; PPARγ, nuclear peroxisome proliferator-activated receptor gamma; ↓, decrease; ↑, increase.

## Data Availability

No new data were created or analyzed in this study. Data Sharing is not applicable to this article.
